# A Unifying Theory for Autism: The Pathogenetic Triad as a Theoretical Framework

**DOI:** 10.3389/fpsyt.2021.767075

**Published:** 2021-11-16

**Authors:** Darko Sarovic

**Affiliations:** ^1^Gillberg Neuropsychiatry Centre, Department of Psychiatry and Neurochemistry, Institute of Neuroscience and Physiology, Sahlgrenska Academy, University of Gothenburg, Gothenburg, Sweden; ^2^Department of Radiology, Sahlgrenska University Hospital, Gothenburg, Sweden; ^3^MedTech West, Gothenburg, Sweden

**Keywords:** endophenotype, framework, genetic architecture, autism model, autism (ASD), exposome, etiology, neurodevelopmental disorder

## Abstract

This paper presents a unifying theory for autism by applying the framework of a pathogenetic triad to the scientific literature. It proposes a deconstruction of autism into three contributing features (an autistic personality dimension, cognitive compensation, and neuropathological risk factors), and delineates how they interact to cause a maladaptive behavioral phenotype that may require a clinical diagnosis. The autistic personality represents a common core condition, which induces a set of behavioral issues when pronounced. These issues are compensated for by cognitive mechanisms, allowing the individual to remain adaptive and functional. Risk factors, both exogenous and endogenous ones, show pathophysiological convergence through their negative effects on neurodevelopment. This secondarily affects cognitive compensation, which disinhibits a maladaptive behavioral phenotype. The triad is operationalized and methods for quantification are presented. With respect to the breadth of findings in the literature that it can incorporate, it is the most comprehensive model yet for autism. Its main implications are that (1) it presents the broader autism phenotype as a non-pathological core personality domain, which is shared across the population and uncoupled from associated features such as low cognitive ability and immune dysfunction, (2) it proposes that common genetic variants underly the personality domain, and that rare variants act as risk factors through negative effects on neurodevelopment, (3) it outlines a common pathophysiological mechanism, through inhibition of neurodevelopment and cognitive dysfunction, by which a wide range of endogenous and exogenous risk factors lead to autism, and (4) it suggests that contributing risk factors, and findings of immune and autonomic dysfunction are clinically ascertained rather than part of the core autism construct.

## Introduction

Autism spectrum disorder (hereafter referred to as autism) is a collective term for neurodevelopmental disorders characterized by impaired social communication, and a rigid and repetitive behavior (1). It is under strong genetic influence (2, 3) and occurs in roughly 1–2% of the general population (4). Its reported incidence has steadily risen in the last decades owing to increased awareness, as well as changes in diagnostic criteria (5, 6).

The autism literature has been particularly fruitful in recent decades; genetic studies have made substantial headway toward identifying contributing variants (3, 7); countless etiological risk factors have been identified (8); endophenotype studies have identified potential biomarkers (9), eventually paving the way for the realization that the underlying features of autism (10), like that of other neuropsychiatric disorders (11, 12), are dimensional rather than categorical. Despite great progress, there are still some outstanding questions meriting intense discussions regarding their theoretical implications and practical relevance.

One of the main scientific and clinical issues is that of heterogeneity (13); behaviorally and biologically, there is no universal risk factor or biomarker that accounts for all autism cases, leading to problems with academic reproducibility and clinical generalizability. It has been argued that the concept of autism lacks validity (14), and that a singular definition should be abandoned (15).

Besides conceptual ambiguity, the lack of a singular explanatory framework that can incorporate findings from across the literature certainly contributes to the heterogeneity. Imagine the heterogeneity of cancer—being caused by risk factors ranging from lack of physical exercise and smoking, to tanning and viral infections, occurring in a wide range of different tissues—before identifying genetic dysregulation of cellular division as its central mechanism. Although the rates and patterns of co-occurring neuropsychiatric conditions (16), sex bias (17, 18), and multitude of risk factors (8) have been well-characterized, the absence of a framework results in a lack of empirical and predictive explanations for the observed patterns.

Several, expectedly overlapping, frameworks have been proposed (see Table 1 for a non-exhaustive list) accounting for various aspects of its pathogenesis. However, none have encompassed all aspects of findings in the autism literature, and many have failed to provide concrete, biologically grounded mechanisms for how these aspects contribute to autism across development and its biological hierarchy. In general, they have been descriptive and lacked operationalization, prohibiting modeling and testing. Even though a unifying theory for autism is lacking, most of the proposed models have significant merit in their own domains, which would complement any top-down unifying theory.

**Table 1:** A selection of models for autism, their explanatory potentials, and relations to the scientific literature.

						**Potential to account for or describe…**									
		**Prescriptive**	**Operationalized**	**Factors and interactions proposed**	**Biological mechanisms explained**	**Genetic architecture**	**Epigenetic mechanisms**	**Biological endophenotype**	**Cognitive endophenotype**	**Behavioral phenotype**	**Clinical phenotype**	**Heterogeneity**	**Sex difference**	**Strengths and abilities**	**Acquired phenotype**
Complex models	Pathogenetic triad (19)	#	#	#	#	#	#	#	#	#	#	#	#	#	#
	Intense world theory (20)	*	#	#	#	#	*	#	#	#	#	*	-	#	-
	Developmental brain dysfunction (21)	#	*	#	*	*	-	#	#	*	*	#	-	-	*
	Neurodevelopmental hypothesis of atypical brain development (22)	*	#	#	#	-	-	#	*	*	*	*	-	-	-
	Dynamic model (23)	#	*	#	*	*	#	-	*	#	*	*	-	-	*
	Uniaxial model (12)	*	-	#	-	*	-	*	*	*	#	#	-	-	*
	Molecular systems-level framework (24)	-	-	#	*	*	*	*	*	*	*	*	*	-	-
	Developmental model of transgenerational transmission of psychopathology (25)	*	-	#	*	-	-	-	-	-	*	*	-	-	-
Simple models	Extreme male brain and empathizing-systemizing theory (26, 27)	#	#	#	#	-	-	*	*	#	*	-	#	#	-
	Excitation/inhibition disbalance (28)	*	#	#	#	*	*	#	#	*	-	-	*	*	-
	Complex information processing disorder (29)	*	#	#	-	-	-	-	#	*	*	*	-	*	-
	Dysconnectivity theory (30, 31)	*	*	#	*	-	-	#	*	#	*	*	-	*	-
	Variable insult model (32)	*	-	#	*	*	*	#	*	*	*	#	-	-	*
	Weak central coherence (33)	#	#	#	-	-	-	*	#	*	*	-	-	*	-
	General psychopathology (34)	#	-	*	-	*	*	*	*	*	#	*	*	-	*
	Mind-blindness (35)	#	#	*	*	-	-	*	*	*	*	-	*	-	-
	Brainstem hypothesis (36, 37)	*	*	#	*	*	*	#	*	*	*	*	-	-	-
	Amygdala theory (38)	*	*	#	#	-	-	*	-	*	*	*	-	-	*
	Enhanced perceptual functioning (39)	*	#	*	-	-	-	*	#	*	-	-	-	#	-
Individual mechanisms	Intellectual disability (40, 41)	#	#	#	*	*	*	-	#	*	*	-	-	-	-
	Growth dysregulation hypothesis (42)	#	#	*	#	*	*	*	*	*	-	*	*	-	-
	Genetic risk – Dominant *de novo* model (43)	#	#	#	*	*	-	*	*	-	*	*	-	-	-
	Risk factors (8, 44)	*	*	*	#	*	*	*	*	-	-	#	*	-	*
	Autonomic disorder (45, 46)	*	#	*	*	*	*	*	-	*	*	-	-	-	*
	Immune disorder (47, 48)	*	*	*	#	*	*	-	*	-	*	*	*	-	-

^#^ and * indicate potential to completely and partially account for, respectively. Prescriptive indicates predictability with regard to diagnosis and prospective outcome. Operationalized indicates existing or possible quantification of model mechanisms. Factors and their interactions are proposed. Biological mechanisms underlying both the factors and their interactions are proposed. The model should also account for the vast array of findings regarding autism, including: the genetic and epigenetic landscapes, such as patterns of common and rare variants, genomic imprinting, and methylation; the biological endophenotype, including biomarkers in both probands and relatives, and effects of risk factors; the cognitive endophenotype, such as low cognitive ability, uneven intellectual profile, increased cognitive ability in relatives, and resistance to optical illusions; the behavioral phenotype, such as rate and pattern of ALTs in probands and extended phenotype in relatives; the clinical phenotype, such as disorder specificity, rate of comorbidities and intellectual dysfunction, and prediction of “loss of diagnosis”; the heterogeneity, both biological and phenotypic; the sex difference, such as biased sex ratio, and differences in ALTs and disease burden; the relationship to strengths and evolutionary adaptations, such as high intelligence and educational attainment in probands and relatives; the possibility of an acquired phenotype after early childhood.

The aim of this paper is to apply the framework of a pathogenetic triad (19) on autism, and exemplify how it relates to the existing literature on autism. The framework consists of three factors (an autistic personality, cognitive compensation, and neuropathological burden) that interact to cause an observable behavioral phenotype which may be identified as maladaptive, sometimes fulfilling criteria for an autism diagnosis. The factors are operationalized, and mechanisms of pathogenesis are outlined. Ultimately, the goal is to synthesize a vast transdisciplinary literature, and account for as many aspects of known findings in autism research as possible. In many respects, it can be complemented by findings and explanations in previously proposed models. A far-reaching goal such as this cannot be completely incorporated into a single framework or paper, and future studies will be needed to specify different aspects of the framework. However, it will hopefully illustrate how a relatively simple and concrete model can account for several findings in the autism literature, as well as propose approaches for solving existing conundrums. Implications and postulates of the framework, and methods for testing and falsification are presented at the end of the paper.

## Brief Outline of Pathogenetic Triad

Fundamentally, the framework proposes three separate and interacting factors:

**1) A common autism core**, conceptualized as an Autistic Personality dimension (AP), although not intrinsically pathological, constitutes an obligate “first hit” for the development of an autistic-like behavior. It is a construct operationalized and represented at different biological levels by a specific genetic architecture, governing the development of a neurobiological and cognitive endophenotype, itself determining the behavioral phenotype.
**2) Cognitive compensation**, conceptualized as Cognitive Capacity (CC), constituting a relative “second hit.” It represents the general ability of the individual to respond to, and learn from environmental cues and demands. It is a construct operationalized as an index of the individual's executive functioning (EF) and intelligence.
**3) Risk factors**, conceptualized as the Neuropathological Burden (NB), constituting an optional “third hit.” It is conceived as the exposure burden to a diverse array of somatic and psychological insults that converge on a common pathway of neurodevelopmental disruption, secondarily inhibiting CC.

**Figure 1:**
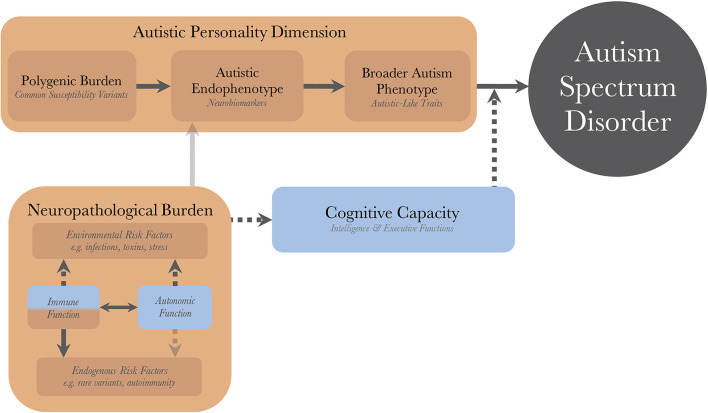
The dynamics of the pathogenetic triad. Presentation of the pathogenetic triad outlining of the interactions between the operationalized factors. Red boxes indicate contributing mechanisms, having positive correlations with severity of clinical phenotype and probability of diagnosis, and blue boxes represent protective mechanisms. Full and dashed arrows indicate contributory and inhibitory mechanisms, respectively. The double arrow illustrates a bidirectional interaction with both contributory and inhibitory mechanisms. Transparent arrows indicate possible mechanisms for which research, with regard to autism, is lacking. There are also gene-environment interactions through other cognitive functions (than cognitive capacity), such as perception and personality, that bidirectionally affect the neuropathological burden, but these have been purposedly left out for model simplicity.

Figure 1 illustrates the pathogenetic triad, and shows how the constituent parts interact to cause an observable maladaptive behavioral phenotype requiring a diagnosis of autism. Briefly, the intensity of the AP depends on the genetic load from common variants and correlates positively with the quantity and intensity of expressed autistic-like traits (ALT), as well as the risk of diagnosis. The AP presents with issues (and strengths) that are qualitatively similar to the diagnostic criteria of autism. These issues are partly compensated for by the individual's CC. For example, high verbal intelligence, theory of mind, and empathizing ability all ameliorate issues with social communication. Similarly, well-developed non-social EFs, such as working memory and inhibitory control, allow individuals to deal with sensory disturbances and digressions from their repetitive and restricted behaviors while maintaining their thought processes, making them appear less “autistic” and more “functional.” Endogenous and exogenous risk factors act as neuropathological insults and disrupt the normal processes of brain and cognitive development. In so doing, they lower the protection offered by the CC, disinhibiting the maladaptive behaviors associated with the AP, sometimes to the point that a diagnosis can be made. There is a disproportionate inhibitory effect on higher cognitive functions (HCF), which themselves are particularly important for compensatory ability. These insults can be alleviated (as a defense mechanism), or caused (autoimmunity and inflammatory reactions), by the individual's immune system, and modulated by the autonomic system; the resulting aggregate effect of this interaction is termed the NB. The heterogeneity in autism, and overlap between different neurodevelopmental disorders, stem from the CC/NB-complex; different individual profiles of CC and neuropathological insults are associated with different neurobiological endophenotypes and behavioral phenotypes within the autism population.

## A Common Autism Core

### The Broader Autism Phenotype

The social, cognitive, and personality characteristics that define autism are also present in neurotypical individuals to a lesser degree without warranting a diagnosis. These characteristics are called ALTs and their presence is referred to as the broader autism phenotype [BAP; see review by (52); also (53–55)]. Autism and ALTs are part of the same common distribution, with the diagnosis merely occurring at the extreme end of that distribution (56, 57). As such, the more ALTs an individual has, the higher the probability of having an autism diagnosis (58).

The importance of the BAP as a concept has gained increasing traction in recent times, and its presence in relatives of individuals with autism has received particular attention (59–63). There is a high heritability, not only for autism, but also for the BAP (64, 65). Studies have shown that relatives of individuals with autism have more such traits than the general population (66, 67), that the degree of genetic relatedness for relatives of diagnosed individuals predicts the risk of them being diagnosed with autism (68), and the degree of BAP in parents predicts some aspects of disease severity in their diagnosed children (62, 66, 67, 69). Multiplex families (more than one child diagnosed with autism) have more ALTs than simplex families (one diagnosed child), which again have more such traits than families without autism diagnoses (60, 61, 69–71).

Given its similarity and overlap with autism, it may offer a window into its core. Assuming it is a phenotype that is shared across the diagnostic threshold (57), one can imagine the BAP to be the defining feature of that which is inherently autistic; its absence will not warrant a diagnosis of autism, no matter the severity of that individual's cognitive impairments or additional risk factors. In other words, it represents the behavioral expression of a core autistic condition (see Box 1 for proposal regarding nomenclature and nosography).

Box 1 **The broader autism phenotype—nomenclature and nosography.**

The existence of a personality dimension which is not intrinsically pathological implicates a normalization of the underlying autistic behavioral pattern. This not only explains the presence of strengths in the autistic phenotype, but is more in line with Occam's razor; postulating a continuous dimensional construct, rather than attempting to explain the presence of the BAP as a categorical extended phenotype in relatives of diagnosed individuals, within a plethora of studies pointing to its polygenic background and normal distribution across the population.

Regarding the nomenclature, rather than recycling the term “BAP-positive” as a binary identifier along a continuous spectrum of ALTs, the term *autistic personality* is introduced to denote the presence of a pronounced BAP. It represents the behavioral expression of an underlying neurobiological endophenotype, which is associated with an increased risk of diagnosis. This is similar to the nosography applied for schizophrenia spectrum disorders (49):

- The autistic personality dimension and psychoticism represent underlying personality dimensions
- Pronounced personality types are identified as an autistic personality and schizotypy
- When these types are maladaptive and give rise to mental health issues, they are denoted autistic and schizotypal personality disorder
- When diagnostic criteria are fulfilled, one receives a diagnosis of either autism or schizophrenia spectrum disorder

Even though individuals with an AP may experience mental health issues, a diagnosis of autism isn't necessarily justified. They will nevertheless be at increased risk of secondary mental health issues such as social phobia, anxiety, depression, and avoidant and restrictive food intake. Long-term outcomes from psychiatric services can be improved if an autistic personality is identified as the cause, such as sensory issues being the reason for food restriction rather than applying a diagnosis of anorexia nervosa. This is particularly true for females with normal or supranormal cognitive ability, who often receive multiple diagnoses and may receive a late autism diagnosis, or altogether fail to fulfill the complete diagnostic criteria for a diagnosis (50, 51).

### The BAP as a Personality Inventory

The pervasiveness of BAP expression across behaviors and the population (72), its normal pattern of distribution (65, 73), its high heritability and polygenic background (74, 75), its stability over time (56, 75, 76), and its potentially beneficial effects from an evolutionary standpoint (72, 77–79) are all hallmarks of a personality inventory [already proposed by Asperger in 1944 (80)].

It is likely that families with a higher genetic burden are right-shifted (increased) on the normal distribution of these traits, leading both to a higher BAP in the non-diagnosed relatives and to a greater familial risk of exhibiting the clinical phenotype which warrants an autism diagnosis [implying a higher incidence in those families; see Figure 1 in (10)].

### The Biology of the BAP

A polygenic nature and population-wide distributions are typical for complex traits (81–83) and common disorders (84). Evolutionary adaptive change is mainly determined by changes in common variants (81, 83, 85), without significant contributions from rare variants (81, 86). The genetic architecture of autism, an arguably complex disease which is associated with strengths, suggests additive effects from both common and rare variants (87–89), where common variants predict risk across the spectrum of disabilities (88, 90), and rare variants are associated with cognitive dysfunction in both clinical and non-clinical populations. For this reason, and reasons that will become evident in sections Cognitive Compensation Protects Against Phenotypic Maladaptation and Risk Factors Affect Neurodevelopment, common variants are postulated to underly the development of a common core autistic neurobiological endophenotype (see section Operationalization of the Autistic Personality), while rare variants will be discussed in section Risk Factors Affect Neurodevelopment as risk factors that influence cognitive development (with at most partial contribution to the core autism endophenotype, in situations where pathophysiological mechanisms overlap).

Neurobiological studies have identified myriad potential biomarkers for autism. Some of them have been identified also in undiagnosed relatives of probands, usually with intermediate phenotypes between probands and neurotypical controls [see reviews by (9, 91)], as expected for a phenotype with a continuous distribution; altered connectivity patterns, excitation/inhibition disbalance, neurophysiological alterations, behavioral and neuropsychological differences, as well as extracranial somatic biomarkers (which are less likely to contribute to the core condition due to lack of specificity; see section Risk Factors Affect Neurodevelopment). Future studies are needed to elucidate which of these endophenotypes may be part of the postulated core autism condition, and which are clinically ascertained endophenotypes resulting from risk factors (potentially offering a route for biological stratification). One telling sign is the indication that common variants converge on synaptic function (7, 92, 93), which may indicate that dysconnectivity and alteration of excitation/inhibition, secondary to synaptic dysfunction, are key biological pathways toward development of the BAP.

Androgens are associated with male behavioral and endophenotypes (94, 95). The increased rates of ALTs (73) and autism (96) among males prompted the conceptualization of the *extreme male brain theory* of autism (26). Increased testosterone may contribute to differences in phenotypic expression [see reviews by (94, 95); also (97, 98); although (99)]. However, it remains to be seen whether the main mechanism of androgens is to directly increase autism risk by increasing ALTs, or if they contribute indirectly through accentuation of male vulnerability through negative effects on social cognition [(97); see section Heritability and Clinical Implications of Low Cognitive Ability] or by modulating biological sensitivity to somatic insults (see section Sex Difference in Biological Vulnerability).

### Operationalization of the Autistic Personality

It is suggested that there exists a common autism core, an autistic personality domain termed the BAP, which is shared across individuals, irrespective of diagnostic status. A pronounced BAP is operationalized as the presence of an AP type. Underlying the AP is a biological hierarchy stemming from common variants. These variants give rise to an intermediate neurobiological endophenotype that ultimately results in a complex trait that we identify as the BAP. The strength of each level along the hierarchy will be positively associated with the BAP and the probability of receiving an autism diagnosis.

The AP is an obligate “first hit” toward receiving an autism diagnosis. A mild personality *de facto* implies a diagnosis of autism shouldn't be made. A pronounced personality may lead to quirks, strengths, and mental health issues, and when the compensatory abilities are low, a diagnosis of autism. Although far from conclusive, genetic and neurobiological studies hint at a core endophenotype that stems from alterations in synaptic function.

The AP can be quantified at each step of the biological hierarchy: common variants may be used to calculate polygenic scores (which should be estimated using ALTs rather than diagnostic status); neurobiological endophenotypes can be identified using brain imaging and neurophysiological methods (which is hampered by biological heterogeneity and difficulty of development of stable biomarkers due to small sample sizes); behavioral questionnaires [such as the Autism Spectrum Quotient (72), or the Social Responsiveness Scale (100)] can estimate the BAP through ALTs. With improved methods and decreased costs, future modeling may allow for the creation of a weighted index from measurements across the hierarchy, further improving clinical utility.

Although individuals with an AP share the phenotypic issues with individuals having a diagnosis, some will not warrant a clinical diagnosis. The reasons some of these individuals develop a maladaptive behavioral phenotype, to the point of requiring a diagnosis, are outlined in sections Cognitive Compensation Protects Against Phenotypic Maladaptation and Risk Factors Affect Neurodevelopment.

## Cognitive Compensation Protects Against Phenotypic Maladaptation

A frequent finding in the literature is the presence of low cognitive ability (intelligence, EF) compared to the neurotypical population [see reviews by (101, 102); also (103–105)]. This is further exemplified by the high rate of clinical co-occurrence of intellectual disability (ID) and autism; 45–75% of patients with autism have co-occurring ID, and 20–40% of patients with ID are found to have autism (4, 103, 106).

Cognitive ability correlates negatively with the number of ALTs being expressed (101, 107), the severity of autism (108), psychotherapy response (109, 110), good outcome (111–115), compensatory ability (116, 117), aspects of social cognition (118), as well as risk of diagnosis (101, 119) and comorbidities (120), illustrating a significant modulatory function of the observed phenotype.

Within the domain of cognitive functions, there is a disproportionate disruption of HCFs, and their impairment seem to be the norm rather than the exception (29, 101, 102, 104, 105, 108, 118, 121–126). Methodological difficulties with operationalization and testing of HCFs exist (105, 122), which likely explains some discrepancies within this field. However, given the protective and enabling effect of HCFs on autism (116, 117, 127–129), it is not surprising that they are often reported to be low in the clinical population.

This pattern of findings may also explain the presence of clumsiness in autism (130), where fine motor coordination is more affected than gross motor coordination (29, 131). Clumsiness and coordination disorder correlate with low cognitive ability (132, 133), also in neurotypical controls and other neurodevelopmental disorders (134), and controlling for cognitive ability removes that association (135). It is possible that suboptimal coordination of neural ensembles in the frontal lobes and across higher cognitive areas gives rise to higher cognitive dysfunction, and that suboptimal neural coordination in the motor systems may give rise to fine motor dysfunction, which, if more pronounced, leads to gross motor dysfunction. Potential reasons why HCFs and fine motor control are disproportionately affected is outlined in section Risk Factors Affect Neurodevelopment.

The presence of an AP induces its own set of behavioral difficulties, such as misinterpretation of social cues and sensory disturbances, that both create issues of their own, and lead to an increased cognitive load. The HCFs enable an adaptive response to such difficulties, as well as to environmental demands [see (129); also (136) for outline of social information-processing mechanisms, and (137) for iterative reprocessing model]. That is, for an adaptive response to a complex environmental demand, such as flexibility in response to a malleable social environment, as opposed to reacting to a sound, the HCFs are instrumental. The compensation likely occurs through both volitional [e.g., IQ and EF facilitating conscious learning of social communication, or through camouflaging; see (129)] and non-volitional cognitive mechanisms [e.g., greater EF allowing for simultaneous processing of sensory stimuli and internal thought processes, or through neural remodeling; (138)].

### Clinical Ascertainment of Low Cognitive Ability

The lower the cognitive ability in general, and the HCFs in particular, the lower the ability will be to adequately respond to the environment and to learn from social interactions (139). This will cause the autistic behavioral phenotype to become, not only more pronounced and easily identified, but also more maladaptive. This increases the probability of being diagnosed, and decreases adaptive ability and long-term prognosis.

The disproportionate disruption of the HCFs can be viewed as particularly unfortunate. However, the flip side is that it can be viewed as telling of the underlying pathogenesis of the clinical phenotype; its presence is expected when viewed through the lens of an ascertainment bias (40). Individuals with an AP that also have a low cognitive ability (relative to their AP) will be more likely to seek psychiatric services and receive a diagnosis.

### Heritability and Clinical Implications of Low Cognitive Ability

Cognitive ability is highly heritable (140–142) with polygenic inheritance [(142, 143); which, as for the BAP, shows convergence on synaptic function, potentially explaining the relationship between polygenic burden for autism and high intelligence (78)]. Its heritability explains why parents and siblings of children with lower functioning autism also have low cognitive ability on average (144–147), but not parents of the subgroup of high-functioning autism (148). Despite the strong clinical connection to low cognitive ability, the BAP and intelligence have been shown to be genetically distinct (149, 150) [for clinical correlate see (111)].

This leads to two conclusions: (1) rather than the AP and low cognitive ability categorically co-occurring (being biologically coupled), a likely explanation is that the distribution of cognitive ability in the clinical population is left-shifted toward lower abilities, and (2) the presence of cognitive dysfunction represents a risk factor, a “second hit,” for developing and expressing a maladaptive behavioral phenotype that we identify as autism because of lower compensation. The idea of reversing the chain of causation is not new (40, 41, 54, 151). A possible causal effect of IQ on the development of schizophrenia (152) has been identified; given similarities in risk factors and cognitive dysfunction across the disorders, the causality may hold true for autism as well.

It is thus possible that the BAP and cognitive ability are distinct phenotypes, and that the presence of a clinical ascertainment bias accounts for the frequent finding of low cognitive ability in autism. Supporting this are numerous studies indicating loss of an association with autism following control of IQ as a confounding variable [co-occurrence of epilepsy (153), coordination problems (135), cognitive abilities in presence of rare genetic variants (154)].

### Operationalization of Cognitive Capacity

CC is conceptualized as the general mechanisms that allow the individual to compensate for, and overcome issues that arise from the presence of an AP. A decreased CC causes the underlying autistic phenotype to become maladaptive, sometimes to the point of requiring a diagnosis. The main components include intelligence and EFs [similar to (29, 129)]. CC can be quantified through the use of various neuropsychological tests.

This framework makes no assumptions about the relative importance of particular subdomains of cognitive ability, but merely acknowledges that (1) some have a greater protective effect regarding the autistic phenotype than others [such as HCF compared with lower cognitive functions, or social cognitive ability and verbal intelligence being better predictors of adaptive ability and optimal outcome than non-social cognitive ability and inhibitory control (129, 155)], and (2) that individual subdomains likely have different effects on different aspects of the phenotype (such as social cognitive ability and verbal IQ having greater effects on Theory of Mind deficits than non-social cognitive ability or non-verbal IQ). It is the task of future neuropsychological studies to elucidate the relative importance of specific cognitive subdomains. One can imagine that a cognitive index with different weighting for each subdomain (which may differ between sexes, see Superior Social Cognition in Females) can be used to improve disorder modeling and diagnostic resolution when employing the pathogenetic triad.

It should be noted that this conceptualization relies heavily on compensation by way of cognitive abilities in order to emphasize the connection to findings of low IQ and EF, and effects of risk factors. However, the concept of compensation in autism is both wider than outlined in this paper, and its presence is not unequivocally positive [see review by (129)].

### Superior Social Cognition in Females

One can identify certain cognitive subdomains that are intrinsically non-social– spatial intelligence, working memory, inhibitory and cognitive control etc.—as well as subdomains that more specifically aid processing of social information and increase social ability—verbal intelligence, interpretation of emotion and biological motion, social cue interpretation, cooperativeness, social motivation etc. Even though the phenotypic separateness of social and non-social intelligence has long been debated (156, 157), they can be construed as distinct concepts (158–160). The purpose of deconstructing cognitive ability into social and non-social domains lies in presenting a mechanism by which CC may give rise to a skewed sex ratio in autism [similar to that of (27)].

Studies investigating typically developing individuals have indicated that females have, what can be considered, a superior social cognitive ability. Some of the sex differences, in favor of females, include superior recognition of non-verbal social and emotional cues, such as faster and more accurate recognition of facial expressions and bodily emotions, more sharing, turn taking and cooperative behavior, being more prosocial, sympathetic and empathetic, and having a higher social motivation [see reviews by (161–163)]. Given the seeming distinctness of BAP and cognitive ability (149, 150), as well as social and non-social cognitive domains (158, 159, 164), it is likely that these sex differences hold true, not only for the NT population, but also for those with autism (163).

The presented sex differences are far from universal, but it is clear that a sex discrepancy within the domain of social cognition exists. On the one hand, females on average show greater social competence. On the other, they are more likely to seek exposure to social interactions, either due to greater inherent social motivation or a lower rate of unsatisfactory and deterring attempts from such interactions, further increasing that competence. Over the course of a lifetime, the difference in social learning and adaptive ability may lead to different life trajectories and subsequent differences in observable clinical phenotype [at least in the subgroup with higher cognitive ability, which has the highest sex-ratio bias (165, 166)].

The distribution of compensatory ability of females may thus be right-shifted (increased), leading to a negative clinical ascertainment bias. Considering a certain base rate of identification (psychiatrists having a set internalized threshold for applying an autism diagnosis), the presence of a protective compensatory mechanism predicts an increased disease burden in other domains. In other words, improved social cognition and adaptive functioning may underly the female protective effect (167) and explain why diagnosed females have lower average cognitive ability than males (168, 169), even when controlling for their lower average BAP (170), or a higher disease burden (107, 166).

In the presence of an underlying autistic phenotype, the cognitive compensation may or may not be complete (129, 155) and can lead to psychiatric problems (171) either due to inadequately compensated social difficulties and a discrepancy between social competence and motivation for social interactions (which may be particularly strong for females), or as a consequence of delayed or undiagnosed autism in females (50, 129, 172). These issues illustrate the need for improved detection of autism in females. If there is a discrepancy in social cognitive ability between the sexes, and it underlies the female protective effect, its inclusion in disease modeling (through sex-specific weighting of social subdomains for cognitive ability) may improve diagnostic resolution for females when applying the pathogenetic triad for identification purposes.

### Relationship Between the Autistic Personality and Cognitive Capacity

**Figure 2:**
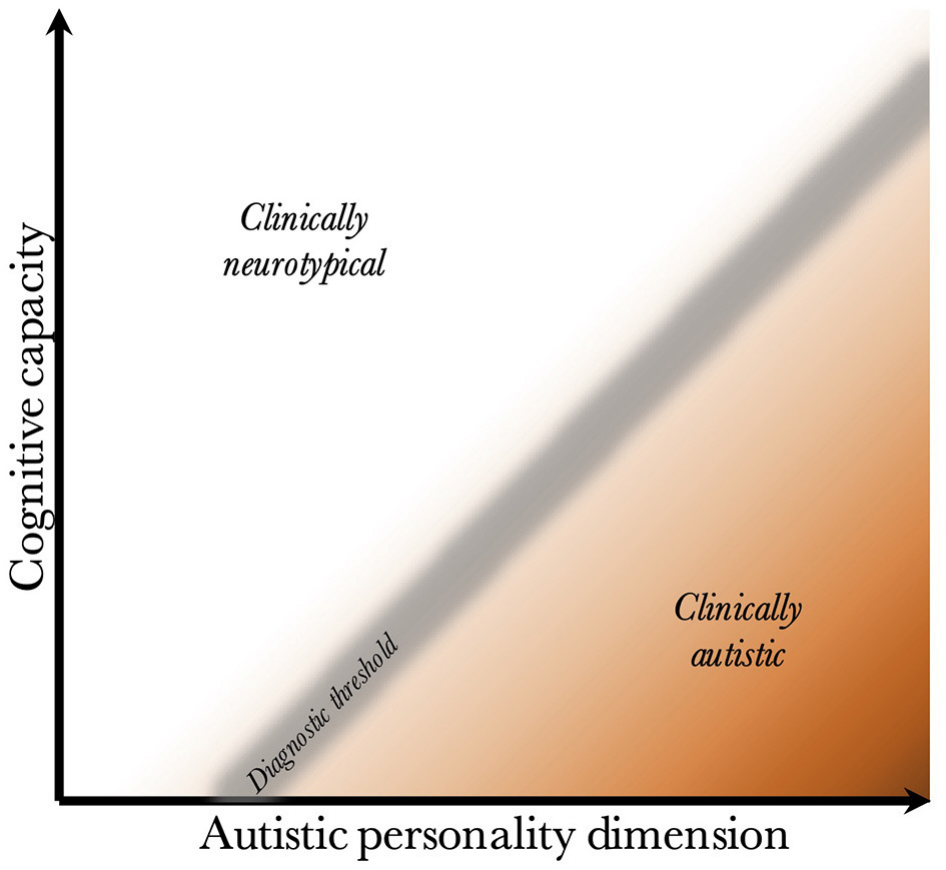
Relationship between cognitive capacity and the autistic personality dimension. This figure illustrates why a higher cognitive capacity, and thus ability to compensate for various behavioral issues, decreases the probability that the intensity of the autistic personality is behaviorally maladaptive. The diagnostic threshold separates the clinical phenotypes into those that are clinically neurotypical and those that fulfill diagnostic criteria for autism. It is a fuzzy demarcation due to the difficulty in determining diagnostic status in borderline cases. The shading below the threshold corresponds to the severity of the clinical phenotype, the probability of having attracted clinical attention and received a diagnosis, and the stability of the diagnosis over time. The true diagnostic threshold is not necessarily straight, but is likely somewhat parabolic and slightly levels off with increasing cognitive capacity.

**Figure 3:**
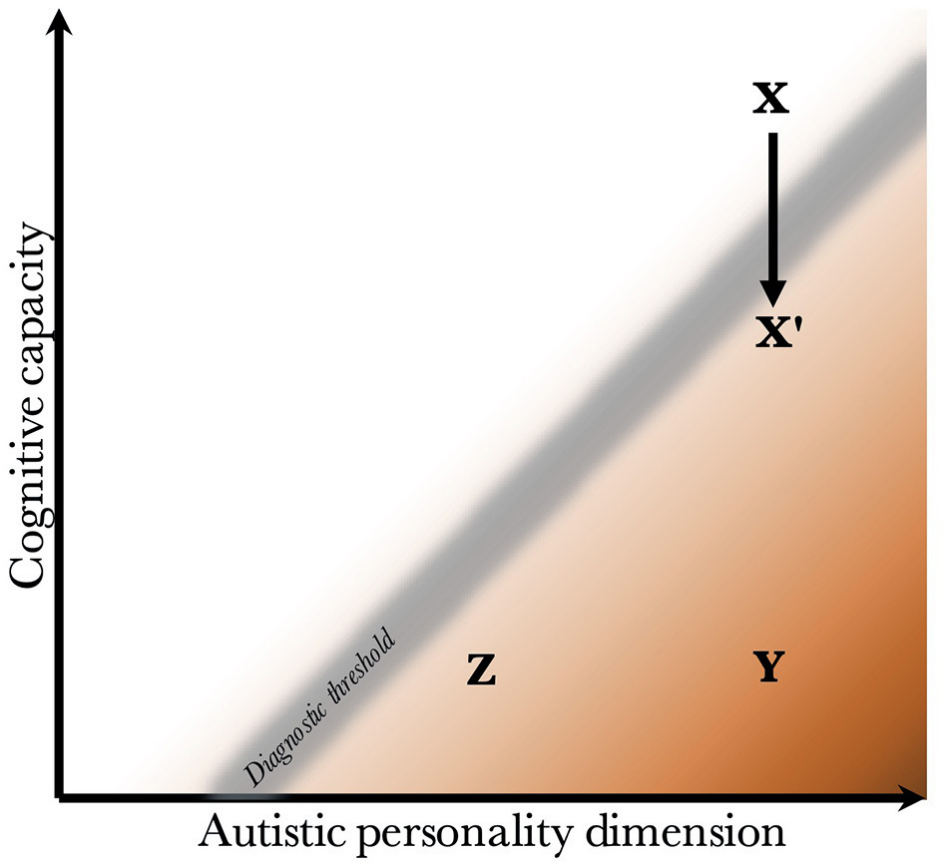
Examples of individuals with varying degrees of cognitive capacity and autistic personality. Presentation of three individuals and their observed phenotypes. Individual X has a high cognitive capacity, which fully compensates for the presence of a pronounced autistic personality. Although X may be perceived as socially odd, no diagnosis is warranted since X is above the diagnostic threshold. Individual Y has an equally pronounced autistic personality. However, the cognitive capacity is much lower, and doesn't allow for adequate compensation of its autistic features. Y fulfills diagnostic criteria, and likely has a moderate to severe autism (long distance to diagnostic threshold). Individual Z also has a low cognitive capacity, which is unable to compensate adequately. However, Z has a milder autistic personality than Y, leading to a milder autistic phenotype (smaller distance to the diagnostic threshold). Finally, X' represents individual X following a neurological insult that negatively affects cognitive capacity. One can imagine X being conceived by healthy parents with a genetic architecture that supports high cognitive capacity and a pronounced autistic personality, but having experienced a perinatal complication or an insult during childhood. The insult lowers the cognitive capacity enough to impair compensation, leading to a diagnosis of autism.

Given that CC and the AP are biologically uncoupled, and that their interaction determines the behavioral phenotype, one can construct a two-dimensional space, which is presented in Figure 2. Since the severity and risk of diagnosis are negatively associated with CC, the phenotypic space is transversed by a diagnostic threshold (DT) with a positive slope (whose true shape is likely slightly parabolic, but is illustrated straight for simplicity). Individuals above the threshold do not have a maladaptive behavioral phenotype and do not require a diagnosis. Individuals below the threshold have a CC that is low relative to their AP, meaning they are not able to compensate for their ALTs. This leads to a maladaptive phenotype requiring a clinical diagnosis. The DT is not well-demarcated due to the difficulty in assigning a categorical threshold to a dimensional construct. The distance to the DT, illustrated by the shading, indicates how maladaptive and severe the observed phenotype is [functional levels according to DSM-5 (1)]. It will also relate to the stability of the diagnosis over time. Figure 3 presents an example of three individuals (X, Y, and Z) with differing degrees of CC and AP, and how that affects their observed behavioral phenotypes.

The figure offers empirical explanations for some findings in the literature. First, it has been found that individuals with higher cognitive ability are more likely to achieve optimal outcome, and lose their diagnosis (no longer fulfill criteria) during future neuropsychiatric evaluations (113–115). This is illustrated by the lower average distance to the DT in groups with higher CC. One can appreciate that within the range of CC for X', the average distance to the DT is much lower than within the range of CC for Y and Z.

Second, it illustrates the counterintuitive finding that diagnosed individuals with a higher IQ have greater deficits in the social domain than those with low IQ (151, 173). This is because the average AP is more pronounced the higher the CC, due to the positive slope of the DT.

Third, although cognitive abilities are temporally stable (in the absence of neurological insults, see section Risk Factors Affect Neurodevelopment), due to changes in stress and cognitive demands throughout life (such as moving to a higher educational level or getting a new job), individuals will experience variable degrees of compensatory ability and thus slightly jitter along the y-axis (similar to movement from X to X' in Figure 3, which is detailed in section Acquired and Atypical Autism, although less pronounced). This exemplifies why individuals with a milder phenotype are more likely to lose their diagnosis. It also illustrates why there is lower diagnostic stability under the autism spectrum disorder umbrella, than there is for autism as a whole [see review by (174); also (175)]; the probability of moving across a single DT is lower than moving across multiple hypothetical thresholds under the DT.

## Risk Factors Affect Neurodevelopment

Within the field of autism research, studies of its causes have increased significantly in the last decades (176). Many different risk factors have been identified [see reviews by (8, 177–179)] and everything from heavy metals and air pollution (8, 178, 180), vitamin deficiency (181), and medications (182, 183), to infections (8, 178, 184), immune disturbances (47, 178, 184–186), pregnancy-related stress (187, 188), and pre- (189, 190) perinatal complications (189, 191) have been noted to increase the risk for autism, with no single cause being specific for the disorder or accounting for a majority of cases. The heterogeneity of autism and its associated risk factors has hampered the development of a unitary pathophysiological pathway. However, since autism is a neurological condition, it is logical that the etiologies show physiological convergence through their effects on brain development.

### Neural Insults Undermine Brain Development

Brain development is a complex and delicate process. Perturbations resulting from biological insults inadvertently shift resources from growth and development, to defense and repair (192), possibly limiting cognitive development in a dose-response manner. There are many examples of the negative effects on cognitive development by risk factors, and similar effects have been found not only for autism (section Risk Factors Affect Neurodevelopment), but also in neurotypical individuals [see Table 2 in (19)]. For example, air pollution (196, 197), vitamin deficiency (198), medications (199), infection (200, 201), immune disturbances (200, 202, 203), pre- (204, 205), and postnatal stress (206–208), and pre- and perinatal complications (209–212) are just some examples of insults that have negative effects on the development of cognitive abilities also in neurotypical individuals.

**Table 2:** Insult characteristics and their effects on the neuropathological burden.

**Insult characteristic**	**Effect on neuropathological burden**
Magnitude	Stronger insults are associated with greater increases in the resulting burden (193)
Timing	Although earlier insults may compensate by gross remodeling, they have down-stream effects on higher cognitive functions and increase burden (193–195). Also, insults occurring during the development of a structure or function are associated with greater burden than after maturation or before their development has begun (193, 194)
Neural specificity	A more specific effect on the central nervous system is associated with a greater burden (such as copy number variants with expressivities in the brain as opposed to other tissues)
Autistic personality-specificity	Insults with physiological mechanisms that overlap with those underlying the development of the autistic personality are associated with a greater burden (such as common and rare variants both converging on synaptic function)

Rather than identifying only very clear insults with large effects [such as valproate exposure during pregnancy, or the presence of genetic syndromes and copy number variants (CNV)], one can imagine that neural insults are normally distributed in the strength of their neurodevelopmental inhibition and thus in their effect on cognitive development. It is near impossible to quantify the detrimental effect of a single mild infection on the individual level, due to miniscule effects on future attainment of cognitive ability. However, one can conceptualize a neural exposome (213, 214) that takes into account all such exposures (stress, toxin exposure, infections etc.), which across an individual's development affect brain development and attained cognitive ability. The *psychopathology-factor* (34) likely correlates with the extent of the conceptualized exposome, and illustrates that it is shared across neurodevelopmental disorders.

The HCFs are disproportionately affected by insults (215). A likely explanation for the discrepant effect is that HCFs and fine motor coordination rely on the coordination between diffuse brain areas and require larger and more complex neural networks (142, 215, 216). This offers many more potential vantage points for disruptions to cause impairment and affect output. Lower cognitive functions and gross motor coordination, on the other hand, rely on localized brain areas and simpler neural networks, limiting the effects of detrimental insults and explaining the faster and improved recovery for these cognitive functions, as well as the possibility of neural remodeling recovering the function. Insults are associated with impaired motor coordination (217, 218), and motor dysfunction relates to cognitive ability also in neurotypical controls and ADHD [Attention-Deficit/Hyperactivity Disorder; (217, 219)], supporting the notion that motor dysfunction may not be a core feature of autism, but a marker of cognitive dysfunction.

Recovery following neurological insults in childhood is less complete than previously thought (193). This may be due to disproportionate effects on HCFs (215, 220) which are difficult to measure reliably and their importance differs across developmental stages (220). It has long been thought that the plasticity of young brains allows for reorganization and catch-up development following insults, a notion that newer studies with longer follow-up and improved methodologies seem to contradict (193, 221). Although it is established that strong insults are associated with low cognitive ability, the difficulty of identifying subtler disruptions of HCFs, and that their importance increases throughout childhood and adolescence, likely explains why milder insults have not received the attention they deserve.

### Genetic Contributions to Risk

As mentioned previously, the genetic architecture of autism implicates both common and rare variants (88, 222) [for presentation of genetic models see (2)]. Rare variants may be inherited or occur *de novo*, and their rate is increased in autism (223, 224). The most common (225) and well-studied types of rare variants in autism are the CNVs (43, 225–227). Rare variants are associated with increased cognitive dysfunction (88, 154, 224, 228–231), disorder severity (224), and rate of comorbidities (88, 166) in individuals with autism. They are also associated with cognitive dysfunction in neurotypical individuals (154, 228, 230). The association between an increased rate of rare variants and autism disappears when controlling for IQ (154), indicating that they may increase autism risk through negative effects on cognitive ability. The prevalence of rare *de novo* variants in the neurotypical population is lower than in autism (43, 229), despite a stochastic distribution across the population (232, 233), further supporting their role in inducing a clinical ascertainment bias.

Several genetic syndromes show increased rates of autism [see review by (234)]. The increased co-occurrence of autism and genetic syndromes may be largely explained by negative effects on CC (234). In other words, the degree of cognitive dysfunction predicts the rate of autism. Which individuals with genetic syndromes ultimately develop autism may depend on the presence of an underlying AP (231). Social dysfunction (235) and a greater polygenic burden for autism (236) in parents predicts the risk of autism in offspring with genetic syndromes, suggesting additive heritability between the AP and NB/CC-complex (sum total of interaction between NB and CC). Additive heritability also predicts higher rates of *de novo* variants in simplex families (43, 229).

Also supporting the notion of genetic syndromes as additive risk factors is that those with autism and genetic syndromes have less social impairment than those with only autism (237, 238) despite a greater disease burden and likely lower adaptive functioning [similarly counterintuitive as for higher IQ (151)]. This may be explained by the presence of a genetic syndrome as an additional risk factor [same pattern also found for co-occurring schizophrenia (239)], which lowers either the diagnostic threshold (upward movement of threshold on y-axis in Figure 2) or the CC (position closer to the bottom), making the autistic phenotype maladaptive at a lower intensity (milder average AP as a group).

Further suggesting that rare variants cause autism through a modulating variable, rather than directly as a core component, is that they are unspecific and shared among the neuropsychiatric disorders (21, 224, 226, 228, 240–243), which is not the case for common variants (240). In other words, the genetic architecture is such that common variants increase the probability of autism across severities and risk factors (236), while rare variants and genetic syndromes cause disinhibition of maladaptive phenotypes, such as autism, by negatively affecting CC.

Although it is expected that common variants are mutually exclusive between disorders, it should be noted that some studies find slight positive polygenic correlations between the disorders (88, 244). A slight overlap is expected due to ascertainment at the disorder-level, rather than trait-level. This leads to erroneous identification of shared common variants that contribute to the NB/CC-complex (inherited common variants for immune and autonomic dysfunction), rather than the AP dimension.

Epigenetic mechanisms, such as methylation (245) and parental imprinting (246), have also been implicated in the development of autism (2, 93, 226). Some epigenetic markers are related to effects of risk factors (237) through gene-environment interactions. There is also some clustering of both rare variants (247, 248) and epigenetic effects (93, 246) on neuronal signaling and synaptic functioning, possibly contributing directly to the development of the AP. Otherwise, the specific epigenetic machinery in relation to autism development is still largely unknown, making it difficult to specify epigenetic mechanisms within an explanatory framework at this time.

### Immune Function as a Modulator of Exogenous Insults and an Endogenous Risk Factor

Many studies have identified immune dysfunction in autism (48, 186, 249–251). For example, differences in human leukocyte antigens (185), such as specific variants (186, 252) and homozygosity (253), are linked to increased risk of autism.

Although central nervous system infections can act as direct risk factors [see review by (254)], indirect effects through gestational maternal immune activation may be of greater significance (178, 255). Indicating a possible causative mechanism by the inflammatory response is that, although gestational fever is associated with increased risk, it can be attenuated by antipyretics (256). The inflammatory response includes production of a range of inflammatory proteins, such as autoantibodies and cytokines.

Autoantibodies are frequently identified (186, 249, 257, 258), illustrating the connection to autoimmunity (250, 257, 259) and pointing to the importance of both systemic and neuroinflammation in autism (251, 260, 261). Differences in the levels of cytokines have been found (178, 255), which may represent both variations in immune activation, and exposure to environmental agents. Cytokines are particularly interesting for their ability to affect aspects of brain function and development (178, 262), such as synaptic function and HPA-axis activation (263).

Similar to the wide range of identified risk factors, the diversity of specific immune alterations that are implicated in the development of autism means that it is unlikely that a singular alteration or mechanism can be identified. Rather, it may be the case that (1) immune dysfunction is an indirect causative agent through mediation between insults and disability, making individuals with immune alterations less able to defend against the deleterious effects of various insults and that (2) immune activation itself acts as an indirect unspecific insult. Supporting this is that both mechanisms may result in cognitive dysfunction (203, 264, 265), and have been identified in other neuropsychiatric disorders (266). The idea that neuropathological insults and immune dysfunctions are causative factors in the development of an autistic phenotype has been proposed previously (21, 32, 178).

### Autonomic Function as Modulator of Insults

The autonomic system represents the interplay between the external and internal milieus. Environmental and cognitive demands are relayed to the internal physiology for proper allocation of resources, supporting either acute survival (a fight-or-flight stress state) or long-term survival (a rest-and-digest state of growth and repair). Adaptable, fast, and flexible coordination of the sympathetic and parasympathetic branches of the autonomic system (267) is necessary for timely allocation of resources. Any dyscoordination within this process (such as a sustained sympathetic arousal long after termination of a brief stressful situation or infection) leads to suboptimal allocation, and thus impaired growth and development.

Autonomic dysregulation is a frequent finding in psychiatric disorders in general [see review by (268); also (269–271)], and autism in particular [see reviews by (45, 272); also (273) for hypothalamic-pituitary-adrenal (HPA) axis]. A flexible and adaptive autonomic response system can be indexed by a high heart rate variability (HRV) which is negatively related to inflammatory markers (268, 274, 275), HPA axis activation (267, 268, 274), mood disorders (268, 269), and risk of autism (45, 46, 267, 272), and positively related to social cognition (268, 272, 276), fluid intelligence (277), and EFs (272, 278).

Many disease states are associated with a decreased HRV; it may be that autonomic dysfunction in clinical populations is due to an ascertainment bias, representing a causative risk factor through its effect on brain and cognitive development. This is supported by higher functioning individuals with autism not having a disrupted HPA axis, while lower-functioning individuals do (273), and that prenatal HRV is more predictive of later social functioning than birth weight, medical comorbidities, or socio-economic status (279). These findings could signify that it is autonomic function, mediating the effect between insults and immunity, that is the determinant of adult morbidity and adaptive functioning, with potential utility in estimating the effect of the exposome.

### Sex Difference in Biological Vulnerability

Sex differences with regard to the AP and CC have been outlined in sections The Biology of the BAP and Heritability and Clinical Implications of Low Cognitive Ability. There is also a potential sex difference within the NB. As proposed in the female protective effect theory, they have a greater genetic burden, illustrated by increased rates of, and more penetrant, risk factors and rare variants (227, 242, 247). This finding may be explained by the presence of risk-protective factors compared with males, such as milder BAP or higher social cognitive ability.

Males have an increased biological vulnerability compared to females [see reviews by (179, 280); also (193, 281)], which is exemplified by greater pre- and postnatal mortality (282, 283), and inhibitory effect by stress on development of the frontal lobe and EF (208). Studies investigating the sex ratio at birth have found that there are less males being born following maternal exposure toxins during pregnancy [although often at higher concentrations than in the general population, see review by (284)], as well as after floods (285), warfare (286, 287), earthquakes (288), and death within the family (289), which all point to an increased vulnerability for males *in utero*. Proposed explanations include a slower maturation and thus higher vulnerability for male fetuses and toddlers, a higher maternal immune reactivity to the presence of a Y-chromosome (280), and inhibitory, or at least modulatory, effects on immunity by sex hormones and glucocorticoids (193, 290–292). Bearing in mind that the sexes have differential responses to exogenous risk factors (293), it is possible that males, as a group, are statistically associated with a higher vulnerability for a greater range of exogenous insults. If males are more biologically vulnerable, it follows that they have a higher average NB for each insult. This implies that females have a higher threshold for accumulating risk factors, with lower disinhibitory effects on cognitive compensation and thus lower rates of maladaptive behaviors and autism diagnoses.

### Operationalization of the Neuropathological Burden

Since autism is a condition affecting the brain, it is logical that the vast range of etiologies show convergence through a common neuropathological mechanism. The NB is conceptualized as the sum total effect of various insults and the effect they have on brain development. Endogenous or exogenous risk factors that negatively affect normal brain development are likely to negatively influence cognitive attainment, impacting the ability to compensate for various cognitive difficulties, including those arising from an AP. To give an analogy, the interaction between the NB and CC acts like a funnel, where a wide range of etiologies come together and cause neurodevelopmental inhibition, which disinhibits a maladaptive phenotype.

Similar causative mechanisms have been proposed previously [*variable insult model*: (32); *developmental brain dysfunction*: (21)]. Although this framework obviously cannot outline the effects of specific insults on risk increase, it emphasizes their additive effects, and the importance of considering also the vast number of low-intensity insults which individuals are subjected to across development (such as stress, minor infections, physical injuries) rather than just the most deleterious ones (such as genetic syndromes, or intrauterine exposure to valproate and rubella).

An individual's immune function and autonomic regulation determine the extent to which insults affect brain development and the neuropsychological profile. Sometimes they alleviate the insult, as when the immune system fends off an infection; sometimes it is the main cause of damage, as when an activated immune system leads to secondary neuroinflammation.

Whether insults cause an altered immunity, or immune dysfunction increases the effects of insults remains to be completely elucidated; one may expect a combination of both. Either way, similar to that of cognitive ability, it is likely that there is a clinical ascertainment bias, wherein individuals with immune and/or autonomic dysfunction are at greater risk of disrupted development, leading to lower CC and increased vulnerability for autism. Finally, HRV may serve as an indirect indicator of the NB. Future studies will elucidate what role HRV plays in relation to autism development, whether it modulates adaptive ability in the face of an insulting agent, is merely a quantification of previous insults, or both.

### Insult Characterization as a Means of Stratification

Insults that individually have low effects and are cumulatively continuously distributed (such as lifetime stress, and immune activation by minor infections or injuries) are more likely to be shared across the population. Conversely, those that have greater effects and a stochastic distribution (such as genetic syndromes, autoimmune diseases, and major stress or injury) are less likely to be shared. Part of the missing heritability may be explained by failure to account for risk-modifying insults, in particular those that are shared and difficult to measure. However, highly penetrant risk factors are more easily identified, allowing them to be individually characterized. Characterization of insults as outlined in Table 2 may be used for biological stratification and increase homogeneity. This extends a previously presented “bottom-up methodology” [(294); where they found increased homogeneity following stratification by insults] and incorporates it into a global framework.

### Epilepsy as an Example of a Neuropathological Burden

Epilepsy is a frequent co-occurring condition in autism (4, 295). Although epilepsy is also a heterogeneous disorder, there are indications that the genetic architecture implicates common genetic variants (296). This is telling of an underlying continuous distribution in the population and predicts the existence of subclinical epileptic activity without seizures. Similar to the BAP and ALTs, this may suggest the existence of a broader epileptic endophenotype, where spontaneous discharges have an underlying normal distribution pattern, with the physiological mechanism being differences in the threshold for neuronal activation.

It is unlikely to be a categorical disorder where individuals with epilepsy differ from those with epileptic activity, who also differ from healthy individuals. Rather, there is a continuum of the extent of spontaneous neuronal activity in the brain, where some individuals have a low enough threshold for the epileptic activity to propagate and cause a seizure. A decrease in the threshold for neuronal activation decreases the signal-to-noise ratio, which effectively decreases the absolute amount of information that can be processed in each neural network, which would induce greater relative issues in the large and complex neural networks underlying the HCFs.

Epilepy has been found to occur in as much as 46% of patients with autism (4), compared to about 0.5% of the neurotypical population (297). Subclinical epileptic activity occurs in a larger proportion of both those with [30–76%; (295, 298, 299)], and without autism [1–4%; (300, 301)]. Following the reasoning outlined above, epileptic activity, and epilepsy even more so, are associated with lower cognitive ability (302, 303), with significantly greater impairment of the HCFs (302), resulting in lower social functioning, and greater severity of autism (153, 304). Thus, similar to the proposed reversal of causation for cognitive ability in autism, the decreased threshold in neural activation might be a (at least partial) cause for the lower cognitive ability and HCFs in that population.

Given the frequent clinical co-occurrence, the obvious effect on neural processing, and the similarity of findings regarding CC, epilepsy is presented as an example of a neuropathological insult causing an increased NB: it is a clearly defined neuropathological entity with different locations, extent of activity, and levels of severity (epilepsy with seizure activity confers a greater burden than subclinical epileptic activity without seizures) that can be postulated to negatively impact bottom-up neural processing, with down-stream disproportionate effects on top-down cognitive ability and HCFs.

The likely association between the conditions is such that the presence of epileptic activity represents a NB which disinhibits the behavioral phenotype of autism, both by negatively affecting cognitive ability, and by inducing its own set of cognitive and sensory disturbances. In support of this conclusion, when controlling for IQ, epilepsy has no effect on the severity of autism and ALTs (153) which may indicate that they co-occur clinically due to an interaction with cognitive ability. This lends further credence to the idea of CC and the NB being drivers for the expression of an autistic phenotype requiring a diagnosis, and their co-occurrence being due to a clinical ascertainment bias.

It should be noted that the increased neural activity with epileptic activity likely influences patterns of synaptic budding and pruning. This may overlap with the pathophysiologic mechanism of the AP, potentially explaining the disproportionate association with autism, possibly out of proportion to its negative effect on CC. Future studies will have to investigate this association within the framework of a pathogenetic triad more closely.

## Implications of the Framework

### Clinical Ascertainment Bias

The high heterogeneity of clinical cohorts suggests that some facets are unlikely to be part of the core autistic condition. This framework suggests that the presence of risk factors, due to their effects on CC, increases propensity to seek mental health services, leading to a self-selection of the clinical sample, and thus a bias in sampling. Conceptually, autism may be a single condition with one common pathway, or many conditions with many pathways with phenotypic convergence that is homogenous enough to be identified as autistic. It has previously been argued against the existence of a singular core autistic condition that is common across the spectrum (14, 15), which is reflected in the widening of the diagnostic criteria and employment of the umbrella term “autism spectrum disorder.” An alternative hypothesis, which is argued in this framework, is that there is a singular core autistic endophenotype where risk factors, such as low cognitive ability and immune dysfunction, co-occur clinically due to an ascertainment bias. Such a situation predicts both the heterogeneity and overlap of risk factors between different disorders [shared psychopathology (34)]. The presence of a clinical ascertainment bias is central to the framework, and it has important ramifications regarding the interpretation of research findings and study design [the importance of which has been noted previously (40, 305)].

**Figure 4:**
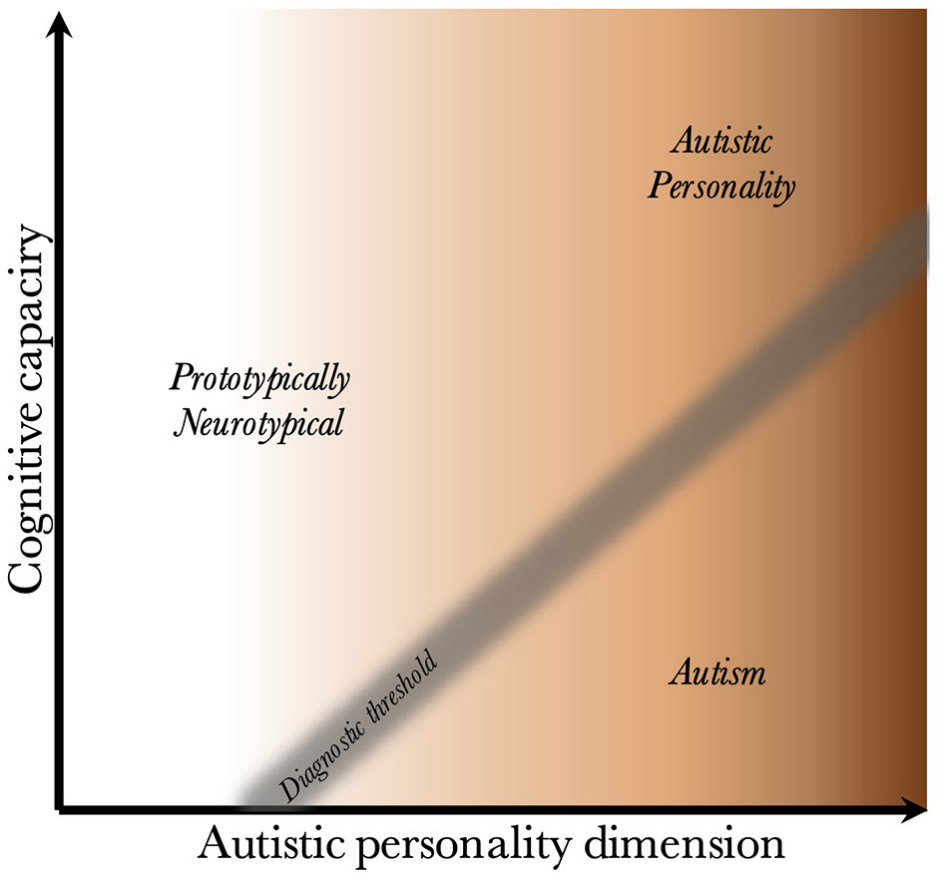
The autistic personality type in relation to neurotypical individuals and those with autism. Illustration how the two clinically neurotypical groups above the diagnostic threshold compare with diagnosed individuals. It shows that individuals with an autistic personality differ from prototypically neurotypical individuals with regard to their position on the autistic personality dimension. It also shows that the difference between having a diagnosis, or not, is contingent on differences in the NB/CC-complex (the result of the interaction between the neuropathological burden and its effect on cognitive capacity).

Although it is precisely the clinical population, with its overlapping risk factors, that is of interest for clinical psychiatric research, for academic inquiry, it may be more fruitful to acknowledge and identify a core autistic condition (assuming it exists), which is presented in this framework as the AP (see Figure 4). For example, when studying risk factors, one wishes to include a control group that differs from the experimental group only with regard to its risk factors; individuals with autism should be compared with a control group that consists of undiagnosed individuals with a pronounced AP (autism vs. pronounced BAP). When studying the development of the core autistic condition, one should include undiagnosed individuals (without risk factors) who differ only with regard to the AP (mild vs. pronounced BAP).

Deconstructing autism into a core condition and associated risk factors may improve study power and predictive ability at the individual level. Population-based samples can limit the ascertainment bias. In studies where such sampling is not feasible, inclusion of undiagnosed individuals with a pronounced AP, for example as a third group within a case-control design, may mitigate the bias and allow for stronger conclusions to be drawn regarding which aspect of autism is affected.

### Stratification

The biological, phenotypic, and clinical heterogeneity of autism presents a great obstacle to its scientific inquiry, having spurred interest in various forms of stratification. This is a difficult task, as it is yet unclear which phenotypes can be stratified and how, and whether one achieves the best results using neuroanatomical, cognitive, etiological, or other endophenotypes. The promises of stratification include greater homogeneity and power in scientific investigations, as well as personalized and tailored pharmacologic, and psychotherapeutic interventions. Not only that, differences in cognitive phenotype lead to different clinical outcomes (111), which necessitates stratification for optimal identification of individuals at high risk of poor outcome, and guidance of choice of treatment depending on who will benefit from it.

This framework argues that the heterogeneity almost exclusively stems from differences in functions and interactions within the NB/CC-complex; each patient has an individual neuropsychological profile (cognitive heterogeneity), and set of insults [biological heterogeneity, further increasing cognitive heterogeneity (154)], that give rise to a large phenotypic heterogeneity. Compounding these with normal variations in personality (outside the AP, such as extroversion/introversion), one can appreciate why no two individuals with autism are alike. A similar explanation has been proposed previously (32).

Unfortunately, most etiologies underlying the NB cannot be identified or quantified; those that can, may be used for stratification through a bottom-up approach for characterization of identifiable discrete insults (Table 2) (19, 294); those that cannot, may be continuously quantified using HRV as a tentative measure of the NB (though, this needs to be specifically addressed and tested in longitudinal studies). Finally, rather than stratifying cohorts according to known insults (which is both difficult and greatly limits sample sizes), employment of the study designs outlined above may be a more feasible approach toward achieving sample homogeneity.

### Heritability and Familial Clustering

All three factors of the triad are heritable (74, 141, 306), with at least partly polygenic inheritance patterns (74, 143, 307, 308) predicting continuous distributions in the population. This illustrates the importance of considering parent phenotypes for risk stratification (231). For example, one can compare the parents' triad mean scores for individual risk stratification, since some individuals will have a starting point closer to a clinical diagnosis than others, irrespective of the presence of risk factors (230). The heritability implies that both ALTs, and idiosyncrasies of cognitive, immune, and autonomic function cluster in families, and in particular in multiplex families. It predicts that multiplex families have higher rates of ALTs than simplex families (60, 61, 70, 71), and that multiplex families have increased rates of rare inherited variants, while simplex families have higher rates of rare *de novo* variants in probands (43, 229). The heritability of the triad not only explains patterns of familial clustering of autism and its associated risk factors, but also the rate of comorbid neurodevelopmental disorders.

### Relationship to Comorbidities

Individuals with a dysfunctional NB/CC-complex (inherited or acquired) will have greater difficulty compensating for the issues arising from the AP. Considering the symptoms of other neuropsychiatric disorders as also being part of dimensional personality types—such as schizotypal traits for schizophrenia spectrum disorders—it follows that an impaired compensatory ability will increase the probability (decrease threshold) that those traits become maladaptive. The lower the threshold, the greater the probability that any, and therefore several, of the phenotypes are pronounced enough to be clinically identified and warrant a diagnosis [as illustrated in Figure 2 in (231), though not only for ID]. This is exemplified by samples with more rare variants (88) and lower cognitive ability having higher rates of psychiatric symptoms and diagnoses, and that ID is the greatest risk factor for the presence of comorbidities (102, 120).

The clinical utility of this is that one can imagine the characterization of an individual's personality profile (pattern of intensities of each disorder-specific broader phenotype) determining which phenotypes are likely to become maladaptive, and that the degree of NB/CC-complex dysfunction determines the probability that each phenotype is maladaptive, and thus the number of diagnoses. Unfortunately, one can expect that the quantification of the effect that the NB/CC-complex has on each disorder is complicated by the fact that the weightings on different insults and cognitive subdomains may be somewhat disorder specific (Table 2), which will require specification through further studies.

Although the case was made above for splitting with regard to academic inquiry, the presence of shared NB/CC-complex dysfunction in clinical populations, due to an ascertainment bias, speaks in favor of lumping in a clinical context. Individuals with lower CC are expected to have greater difficulty across phenotypes, and are thus more likely to require help from psychiatric services. Prognosis and long-term outcome are often highly dependent on cognitive and adaptive ability (111, 115, 309) rather than on the strength of the underlying phenotype. Not fulfilling complete criteria for a single disorder does not rule out the possibility of subthreshold problems from several domains, which may still be associated with significant disability (21, 231, 310). There is likely a common underlying psychopathology that needs to be taken into account when assessing neuropsychiatric patients (21, 34); given its importance for disorder severity and long-term prognosis, its characterization should perhaps be given higher priority. The AP may be particularly illustrative, since individuals without NB/CC-complex dysfunction may lead fulfilling lives and have successful careers despite having pronounced ALTs (72, 311).

### Sex Difference

The male-biased sex-ratio has steadily fallen due to improved detection and awareness of autism in females. However, potential sex differences due to differences in sex hormones exist in all three factors of the triad: females have a lower BAP, higher social cognitive ability, and they may be less biologically vulnerable to neuropathological insults. These differences predict that the true clinical sex-ratio is at least slightly male-biased, and that a clinical 1:1 ratio probably indicates overdiagnosis of females with autism.

Proposed theories for the sex difference include the *extreme male brain theory* [which proposes that higher testosterone and a neurobiologically male brain confer a greater risk for autism; (26, 312)], the *female protective effect* [which proposes genetic, or other features that lower their disorder severity, or raise the threshold for diagnosis; (167)], and a *difference in behavioral phenotype* between the sexes (313).

Neuroendocrinological differences, primarily due to lower testosterone, give rise to a milder BAP and higher social cognitive ability. This combination may lead to the emergence of different compensatory strategies and cause the clinical appearance of females to present with a different phenotype. Given a compensatory mechanism (such as a lower BAP, a higher social cognitive ability, or a lower biological vulnerability for insults), a higher disease burden is required for the same observed phenotypic expression and rate of diagnostic detection. In other words, the *extreme male brain theory* and *difference in behavioral phenotype* can explain the occurrence of the increased disease burden proposed within the *female protective effect* theory (at least within the clinical population). All three theories have supporting evidence, indicate that they all, to some extent, contribute to the sex differences. The presented framework instead attempts to offer one common conceptualization incorporating all three theories.

### Loss of Diagnosis

In longitudinal follow-up studies, some individuals fail to fulfill the full diagnostic criteria, and thus lose their diagnosis. This is not to say that they are less “autistic” since the BAP is highly stable across time, even in individuals with a diagnosis (56). However, they can appear to be less maladaptive, thus not requiring a formal diagnosis. Studies indicate that this “loss of diagnosis” occurs more often in individuals with higher cognitive ability (114, 314), which is empirically predicted by the patient population having a lower average distance to the diagnostic threshold among those with higher cognitive ability (due to the positive slope of the threshold; see Figure 2). With regard to how dynamic changes in the psychosocial environment may lead to differing demands, affecting adaptive and maladaptive phenotypes, the pathogenetic triad can be supplemented by explanations in the dynamic model (23).

### Acquired and Atypical Autism

The proposed framework opens the possibility for an acquired phenotype even beyond childhood; a drop in CC relative to the AP may induce a maladaptive phenotype warranting a diagnosis (X → X' in Figure 3). There have been reports of acquired autism following early childhood (315–317). However, parental BAP or premorbid BAP in the proband were not reported. They may have already had increased liability through a more pronounced BAP. Future studies on acquired autism should include this aspect.

Even in the absence of an AP, it could be possible that impairment of specific functions or areas, such as those subserving social cognition or Theory of Mind (318), can induce a social communication deficit that appears autistic. This will likely not be associated with other patterns that are specific for autism, outlined in this framework, and may underly the presentation of atypical autism or pervasive developmental disorder-not otherwise specified. Transgenerational studies investigating these subgroups' endophenotypes and BAP may shed light on this possibility.

### Strengths and Savant Syndrome

The BAP is associated with a set of strengths (72, 77, 78, 311), and the common variants underlying autism are associated with high cognitive ability and educational attainment (79, 88, 89, 319). These aspects may predict, at its extreme, an increased prevalence of savant syndrome in autism (320).

However, due to the frequent association with cognitive dysfunction in clinical autism, this requires an explanation. There are two possible explanations for these patterns: either autism represents a single endophenotype associated with an inverted U-curve with respect to cognitive ability, as predicted in (20), or the strength-associated phenotype (AP) is uncoupled from the clinical phenotype (autism), as predicted by the pathogenetic triad.

It has been found that patients with severe ID have higher polygenic scores for educational attainment than those with mild/moderate ID (89). Having in mind that this is a single study, this is more in line with the former explanation, and could represent a minor invalidation of the pathogenetic triad. The triad can accommodate this finding if the common variants underlying the AP induce differential susceptibility [decrease resilience and increase plasticity; (321, 322)], leading to increased attainment in enriched environment and lack of risk factors. One should remember that studies identifying an association between polygenetic burden and cognitive ability have primarily used samples from first-world countries. Future studies will have to investigate the possibility of the AP inducing differential susceptibility.

## Discussion

This paper has attempted to outline the pathogenetic triad and illustrate how it relates to the autism literature. The discussion will outline the validity and limitations of the framework and provide examples of testable postulates and hypotheses.

### Framework Validity

“*All models are wrong, but some are useful”*—George Box

It is a fallacy to think one can model a complex and multifactorial disorder, such as autism, using a single reductionist model. One needs to identify an optimal tradeoff between simplicity and specificity, and the validity of the model ultimately depends on the balance of this tradeoff. This framework is no different; specificity has been traded for explanatory power with regard to global pathogenetic mechanisms. The lack of specification means there will always be individual studies that oppose the outlined mechanisms. This does not necessarily undermine the framework, and future systematic reviews focusing on each proposed mechanism and interaction will be instrumental in supporting its validity.

Clinical neuropsychiatric evaluations for autism involve the identification of autistic symptoms and behaviors (AP), neuropsychological testing (CC) and identification of severe and explicit insults, such as perinatal complications, diseases or genetic syndromes (NB). This general methodology implies a collective awareness of (at least) a three-factor model underlying autism, with similar factors as those presented in the framework, which supports the *face validity* of the proposed framework.

Within the autism literature, there are a range of findings that need to be incorporated into a model for autism before it can be considered complete: biological and phenotypic heterogeneity, differences in phenotype and prevalence between sexes, the presence of cognitive strengths in both autism and the BAP, and several cases of acquired autism beyond the usual time of onset. Besides these findings, a complete model must also account for findings regarding its genetic architecture, biological and cognitive endophenotypes, the behavioral phenotype, and clinical correlates such as prevalence rates and co-occurring disorders. As can be seen in Table 1, of the existing explanatory models, this framework is the one that pushes *content validity* the furthest in terms of the number and range of domains of autism research findings that it attempts to incorporate.

*Criterion* and *discriminant validity* implies that the framework, when applied practically, achieves a high diagnostic resolution for the studied phenomenon. The validity of the pathogenetic triad has been examined in a pilot study (323), with classification performed against the participants' diagnostic status based on gold standard neuropsychiatric evaluations and clinical diagnoses. The approach for operationalization outlined in this paper was used for quantification and classification: the autistic personality dimension as the AQ score (72), cognitive compensation as the working memory IQ subscale of the WAIS (324), and the neuropathological burden as the cardiac vagal index (a measure of HRV using electrocardiography). By collapsing the three-dimensional data space onto a one-dimensional axis using linear transformations one was able to achieve a high diagnostic accuracy with an area under the receiver operating characteristic of 96.3% [95% CI (0.913–1.000)]. The method in the pilot study exemplifies how the framework can be practically used, and illustrates its potential for yielding a high diagnostic resolution. The diagnostic odds ratio was 85.5 for the case-control classification which, unfortunately, included only normal IQ individuals matched for total IQ (due to convenience sample); a testament to its high discriminatory power. Since the ratio and AUC both depend on disease severity, one can expect even higher accuracy following inclusion of individuals with lower IQ, and particularly when tested on a sample that has not been IQ-matched. *Divergent validity* was not investigated, as no other NDDs were sampled. However, it is assumed that there is divergent validity due to the specificity of the first factor; substituting for a schizotypal personality type, for example, should by definition yield an unrelated classification (inversely to the degree of overlap between the quantitative measures used to estimate the core conditions, such as the AP and schizotypy). *Convergent validity* implies that constructs that are theoretically supposed to overlap, in fact overlap, such as shared etiologies between different neurodevelopmental disorders. The framework has convergent validity insofar as the NB/CC-complex dysfunction is postulated to be shared among disorders.

The divergent and convergent validities together make up, and support the *construct validity* of the framework. Its construct validity is further supported by the many findings in the literature that fit with the presented mechanisms, many of which (although non-exhaustively) have been outlined throughout the paper, implying that the inferences made from the model reflect the intended construct. Some of the predictions and testable postulates of the framework (Table 3) already have provisional support, while others are yet to be validated.

**Table 3:** Testable postulates of the pathogenetic triad.

1	The pathogenetic triad factors (AP, CC, and NB) are continuously distributed in the population, and the ends of their distributions are disproportionately associated with a diagnosis.
2	The strength of the AP in parents will be positively associated with the prevalence and severity of autism in the next generation.
3	The CC of parents will be negatively associated with the prevalence and severity of autism in the next generation.
4	The distance to the diagnostic threshold (when plotting the AP against CC or the NB/CC-complex) is positively related to severity, probability of diagnosis and diagnostic stability over time. The three-dimensional principal component for the pathogenetic triad will correspond to the clinical impression of the autistic phenotype, from neurotypical to severely autistic, and thus probability of diagnosis.
5	In diagnosed individuals, a more pronounced AP will be associated with a higher average CC, and vice versa, due to the positive slope of the diagnostic threshold.
6	CC negatively correlates with the probability and severity of autism, and the number of neuropsychiatric comorbidities.
7	HCFs are more important for compensation and risk prevention. As such, they are more negatively affected than lower cognitive functions in diagnosed individuals, and their recovery is lower following neuropathological insults.
8	Common genetic variants (polygenic burden) predict the strength of the AP, and are positively associated with the probability of diagnosis.
9	Common variants are specific for ALTs. However, in clinical samples, autism will show minor overlap with other neuropsychiatric disorders due to polygenic heritability within the NB/CC-complex. Thus, the degree of overlap of common variants increases with the number of neuropsychiatric comorbidities in the tested sample and is higher in multiplex families.
10	Rare genetic variants/syndromes, and endogenous/exogenous insults, are associated with a decreased CC.
11	Insults that are associated with the greatest cognitive deficits will be most likely to cause autism. The prevalence of individual insults in the clinical population will be positively related to their effects on brain development. Insults with negligible associations with cognitive deficits will not co-occur with autism more often than in the neurotypical population (except in cases where the pathophysiology overlaps with that of the AP, in which case there is a lower association with CC).
12	Individual insults will be more strongly associated with certain disorders if their pathogenetic mechanisms overlap (such as having an effect on synaptic function and autism).
13	NB/CC-complex dysfunction is shared across neurodevelopmental disorders, and predicts the number of such diagnoses.
14	NB/CC-complex dysfunction causes a clinical ascertainment bias; low CC and the presence of neuropathological insults will prospectively predict who presents to psychiatric clinics.
15	Findings associated with the clinical ascertainment bias (cognitive, immune, and autonomic dysfunction) are minimized in samples with higher cognitive function, and disappear in neurotypical individuals with a pronounced AP.
16	Control for CC eliminates, or lowers, association between autism and associated findings such as immune alterations, autonomic dysfunction, and insults (such as rare genetic variants and perinatal complications) in clinical samples.
17	Different insults are associated with different biological and cognitive endophenotypes.
18	Biological and phenotypic heterogeneity increases with decreasing cognitive ability.
19	Homogeneity is approached through stratification according to specific insults or insult characteristics.
20	Biomarkers that are not part of the biological hierarchy of the core autism condition (AP) will be unspecific across neuropsychiatric disorders. Neural–immune–autonomic–somatic biomarkers are expected to be increasingly less specific for autism and more shared across disorders.
21	Multiplex families will have a more pronounced AP and a higher rate of NB/CC-complex dysfunction than simplex families.
22	Multiplex families will have higher polygenic risk scores for the AP, cognitive impairment, as well as for immune and autonomic dysfunction, than simplex families.
23	Probands in simplex families will have more penetrant risk factors than in multiplex families.
24	Simplex families will have a greater parent-proband difference in CC.
25	Multiplex families will have a higher prevalence of *inherited* variants (as part of a dysfunctional NB/CC-complex), while simplex families will have higher incidence of *de novo* variants.
26	Genetic syndromes will have an autism prevalence inversely related to their average cognitive ability; syndromes with greater average cognitive deficits will predict higher rates of co-occurring autism.
27	For population-based studies on genetic syndromes, parent/sibling AP relates to the probability of an autism diagnosis in the proband.
28	Individuals with a diagnosis that have protective factors (such as higher IQ or lower biological vulnerability) will on average have more risk-increasing factors (more ALTs or rare genetic variants), and vice versa (given a stable base rate of identification—a set diagnostic threshold—such as for single site studies, or single neuropsychiatric evaluators).
29	With the presence of a relatively pronounced underlying AP, insults that negatively affect general cognitive ability, and the HCFs in particular, may disinhibit an autistic phenotype that requires a diagnosis.
30	Insults that negatively affect specific neurobiological structures (for example those subserving ToM and social neural networks), may give rise to an acquired phenotype that appears autistic, but without the other associations outlined in the framework. In other words, acquired autism due to circumscribed damage is less likely to both fulfill all criteria, and to show typical patterns for autism such as heritability, biology and phenotype.

*ALT, Autistic-Like Trait; AP, Autistic Personality; BAP, Broader Autism Phenotype; CC, Cognitive Capacity; IQ, Intelligence Quotient; NB, Neuropathological Burden; ToM, Theory of Mind*.

### Limitations

There are several potential limitations that need to be addressed before this framework can be considered valid. The most important is that the conclusions are supported by a wide, transdisciplinary, and for obvious reasons, non-systematic literature search. The method of identification of relevant literature may have been affected by subjectivity and confirmation bias, and important references have certainly been omitted. This mandates that each aspect of the framework be subjected to individual systematic reviews.

The framework is presented as a top-down model that outlines global mechanisms for pathogenesis. As such, its direct clinical applicability is limited by the lack of specification of the operationalized variables. It merely acknowledges the likely importance of certain subdomains, which can guide development of weighted indices for each factor. Future studies applying the pathogenetic triad, such as classification studies, will indicate which weightings are superior. This will be guided by achieved classification accuracies and potentially illustrate underlying physiological and phenotypic mechanisms.

Although CC is presented as the holy grail of compensation and diagnostic prediction, it is not expected to be the sole determinant of long-term functioning and outcome. First, it emphasizes the relative contributions of different subdomains; some individuals with a very high IQ may still have a diagnosis of autism, likely because of the presence of a much more pronounced AP and/or deficits in some EFs which do not enable adequate compensation. Second, the behavioral phenotype is affected also by psychosocial factors, such as additional mental health issues, personality differences, and sociodemographics. For example, individuals with social phobia and high IQ may have worse outcomes than those without phobia and low IQ. Although left out for model simplicity, psychosocial factors may have to be included as a moderating variable for completeness (in which case they likely moderate, and are moderated by CC in Figure 1).

One can argue against the validity of grouping cognitive abilities into a common category. However, although another conceptualization may prove superior, this approach accommodates the range and pattern of risk factors in the clinical population, and provides a unifying mechanism by which the range of risk factors can induce autism. At least through the prism of an underlying singular core autistic condition. It is also possible that there is no such core condition, and that the autisms are truly as heterogeneous as they seem.

The proposed separation of rare and common variants is based on their co-occurrence with and without cognitive dysfunction, which also suggests differing contributory mechanisms. However, this view of the genetic architecture may be optimistic due to the complexity, and still relatively unknown inner workings of the genetic and epigenetic landscapes (325), requiring further study. In clinical populations, there is clustering of rare variants in parts of the genome associated with development of the brain and cognitive ability (154). Rare variants may occur in locations that overlap with the physiological mechanisms underlying the development of the AP, implying that they may give rise to the AP without affecting the CC. Oftentimes the locations are mutually exclusive, but sometimes they overlap, subserving the development of both the AP and CC; this pattern can be identified and empirically tested by comparing cognitive ability and the AP across genetic markers using large sample sizes.

Many conditions are associated with autism (e.g., ADHD, immune and autonomic dysfunction, developmental coordination disorder) and it is not always clear if they are merely clinically coupled, or inextricable parts of the autistic condition due to genetic and biological coupling. For example, it has been suggested that autism and ADHD are part of a single continuum with a common origin (326), and that autism is an autoimmune disease (47). These conclusions could be explained by a lack of control of extraneous variables, such as a shared dysfunction of the NB/CC-complex due to a clinical ascertainment bias, giving rise to a common endophenotype. Findings supporting a common origin invalidate the presented framework. However, some associations are due to methodological constraints, such as failure to account for extraneous variables. Before such studies can be said to undermine the validity of the framework, their conclusions and methodologies need to be revisited in light of the presented framework.

The biological hierarchy for the BAP, through the effects of common variants, is considered homogeneous for model simplicity. Whether the BAP consists of a single entity on a spectrum (10, 65, 327), or several disparate but, more often than not, co-occurring factors (164, 328) remains an open question. It may be the case that autism does not have a singular susceptibility core, but a combination of endophenotypes that co-occur more often than expected by chance (such as separate susceptibilities for social communication and rigidity/repetitive behaviors). If that is the case, the validity of the framework is not undermined. However, the first factor (AP) would require revision, increasing the complexity of the framework. In the proposed conceptualization, for quantification purposes and clinical testing, one can consider the BAP to be a single entity, since the absolute magnitude of such traits confers a risk factor; if the traits stem from separate factors, the presence of two rather than one such factor, or the presence of more intense such traits, confers a higher total aggregate risk. If that is the case, one can imagine some factors to be more “detrimental” than others, in terms of probability of requiring a diagnosis (such as social > rigidity/repetitiveness), but to outline their relative contributions is outside the scope of this paper.

### Postulates and Testable Hypotheses

Postulates of the framework are presented in Table 3 as a means of generating hypotheses for future studies, and as a means of enabling falsifiability of the framework. Failure to replicate the following postulates, if not explainable through inadequate study design, may be considered as invalidating the framework.

## Conclusion

A unifying framework for the development of autism is presented through the prism of a pathogenetic triad. The major aims of the framework have been to incorporate a wide range of transdisciplinary research findings, and to increase the practical utility of autism models through operationalization. The theory assumes a top-down approach by focusing on outlining global mechanisms of pathogenesis. There is limited specification at each of the factors and bottom-up studies are needed to delineate the specifics of each factor and their interactions. Despite this, it can still serve to inform reasoning regarding future research, interpretation of findings, and development of classification methods. In its simplest form, ALTs and polygenic risk scores can be collated with data on cognitive abilities (IQ, EF) and insult burden (rare genetic variant burden, insult characterization, HRV, immune/autonomic dysfunction polygenic risk scores) to allow for modeling and testing of its predictive ability on diagnostic status. Biologically grounded classification models may prove instrumental in improving the detection of autism in females and individuals with supranormal cognitive ability, for which the clinical interview has low sensitivity.

Although several aspects of the framework have been individually outlined previously, this is the first attempt at conceptualizing a unifying theory that ties together both previously proposed mechanisms, and the wide range of findings in the autism literature. The following are the main contributions of the framework:

- It is the first attempt to incorporate several patterns of findings in the autism literature into a single model: sex differences, strengths associated with the BAP, biological and clinical heterogeneity, and the possibility of acquired autism after early childhood.
- It presents a convergent pathophysiological mechanism, through inhibition of neurodevelopment and cognitive ability, as an explanation for the wide range of identified risk factors.
- It presents the BAP as the common core autistic condition, representing a personality dimension which is decoupled from the cognitive dysfunction of the clinical phenotype, and not pathological in itself.
- It proposes that low cognitive ability, and immune and autonomic dysfunction are not part of the core, but act as independent risk factors that induce a clinical ascertainment bias. This has implications for its scientific inquiry in general, and for explaining the heterogeneity and clinical overlap among disorders in particular.
- It emphasizes the importance of considering the cumulative effects of low intensity insults on brain development, compensatory ability, and risk of diagnosis.

## Data Availability Statement

The original contributions presented in the study are included in the article/supplementary material, further inquiries can be directed to the corresponding author.

## Author Contributions

The author confirms being the sole contributor of this work and has approved it for publication.

## Funding

The author was supported by Fredrik O Ingrid Thurings stiftelse (2018-00419; 2020-00581), the Gothenburg Society of Medicine and Kristina Stenborgs Stiftelse (GLS-960453), Stiftelsen Professor Bror Gadelius Minnesfond, Stiftelsen Systrarna Greta Johansson och Brita Anderssons minnesfond, and FoUU Department of Radiology, Sahlgrenska University Hospital. Other than financial support, there was no involvement in the paper.

## Conflict of Interest

The author declares that the research was conducted in the absence of any commercial or financial relationships that could be construed as a potential conflict of interest.

## Publisher's Note

All claims expressed in this article are solely those of the authors and do not necessarily represent those of their affiliated organizations, or those of the publisher, the editors and the reviewers. Any product that may be evaluated in this article, or claim that may be made by its manufacturer, is not guaranteed or endorsed by the publisher.

## Abbreviations

ADHD: Attention-Deficit/Hyperactivity Disorder
AP: Autistic Personality
BAP: Broader Autism Phenotype
CC: Cognitive Capacity
DSM: Diagnostic and Statistical Manual of mental disorders
DT: Diagnostic Threshold
EF: Executive Function
HCF: Higher Cognitive Function
ID: Intellectual Dysfunction
IQ: Intelligence Quotient
NB: Neuropathological Burden.
